# Integrated Human Exposure to Air Pollution: A Step Further

**DOI:** 10.3390/ijerph20227061

**Published:** 2023-11-13

**Authors:** Nuno Canha, Evangelia Diapouli, Susana Marta Almeida

**Affiliations:** 1 Centro de Ciências e Tecnologias Nucleares (C^2^TN), Instituto Superior Técnico, Universidade de Lisboa, Estrada Nacional 10, Km 139.7, 2695-066 Bobadela LRS, Portugal; smarta@ctn.tecnico.ulisboa.pt; 2 National Centre for Scientific Research “Demokritos”, Agia Paraskevi, 15341 Athens, Greece; ldiapouli@ipta.demokritos.gr

Along with climate change, air pollution is one of the biggest environmental problems affecting everyone in the world today. According to the World Health Organization, air pollution causes an estimated 7 million premature deaths and reduces the healthy life years of millions more [1]. The burden of disease attributable to air pollution is now thought to be comparable to that of other major global health risks, such as unhealthy diets and tobacco smoking.

In order to reduce the negative impact of human exposure to air pollution on the health and well-being of citizens, it is essential to understand and develop strategies and mitigation measures to control it. However, the exposure of citizens to air pollutants is typically based only on the concentrations of pollutants measured at air quality monitoring stations operated by national environmental agencies. These monitoring stations focus on outdoor air quality and are usually located in urban centres.

This approach does not take all components of exposure into account, as people spend a large proportion of their time indoors and have different time–activity patterns, and there is also high variability in air pollutant concentrations within a given city.

Therefore, human exposure over a whole day cannot be reflected only by outdoor exposure, and should consider all micro-environments wherein people spend their time (e.g., home, workplace, transport, leisure, and others) and the time spent in them. The characterisation of indoor and outdoor environments is essential to assess integrated human exposure to air pollutants.

The need to increase knowledge in this area led us to create a Special Issue of IJERPH dedicated to integrated human exposure to air pollutants (https://www.mdpi.com/journal/ijerph/special_issues/IHETAP, accessed on 1 September 2023). Considering the great interest that this Special Issue has received from researchers all over the world in its first edition, we felt that a second edition would be useful to collect additional valuable information and research that has been carried on this important topic out since then.

With this second edition of the IHETAP Special Issue, we invited colleagues to contribute with research focusing on human exposure in different microenvironments, individual exposure under specific conditions and activities, and methodologies to understand pollution sources and their impact on indoor and ambient air quality, with the main aim of developing effective mitigation measures to reduce human exposure and protect public health.

In total, the second edition of this Special Issue brings together eighteen peer-reviewed open access articles that provide new insights into important topics in the field of human exposure to air pollution. Overall, five main areas have been discussed and explored in this Special Issue, namely (i) new methodologies for human exposure assessment (contribution 1); (ii) citizen empowerment with regard to air pollution (contributions 2, 3, and 4); (iii) outdoor air pollution (contributions 5, 6, 7, and 8); (iv) indoor air quality (contributions 9, 10, 11, 12, and 13); and (v) the health effects of human exposure to air pollution (contributions 14, 15, 16, and 17). We highlight two review articles in this edition of our Special Issue that provide comprehensive understanding of the impact of climate change on indoor air quality (contribution 12) and the health effects of long- and short-term exposure to ambient NO_2_ and PM_2.5_ (contribution 18). We believe that these studies will make a significant contribution to the advancement of knowledge in this field.

As editors of this Special Issue of IJERPH, we would like to acknowledge the great efforts of everyone involved in this process, from the dedication of all the reviewers who provided valuable feedback to the authors and the editorial team.
